# Identification of self-interacting proteins by integrating random projection classifier and finite impulse response filter

**DOI:** 10.1186/s12864-019-6301-1

**Published:** 2019-12-27

**Authors:** Zhan-Heng Chen, Zhu-Hong You, Li-Ping Li, Yan-Bin Wang, Yu Qiu, Peng-Wei Hu

**Affiliations:** 1 The Xinjiang Technical Institute of Physics and Chemistry, Chinese Academy of Sciences, Urumqi, 830011 China; 2 University of Chinese Academy of Sciences, Beijing, 100049 China; 3 IBM Research, Beijing, 100049 China

**Keywords:** Self-interacting proteins, PSSM, Random projection, Finite impulse response filter

## Abstract

**Background:**

Identification of protein-protein interactions (PPIs) is crucial for understanding biological processes and investigating the cellular functions of genes. Self-interacting proteins (SIPs) are those in which more than two identical proteins can interact with each other and they are the specific type of PPIs. More and more researchers draw attention to the SIPs detection, and several prediction model have been proposed, but there are still some problems. Hence, there is an urgent need to explore a efficient computational model for SIPs prediction.

**Results:**

In this study, we developed an effective model to predict SIPs, called RP-FIRF, which merges the Random Projection (RP) classifier and Finite Impulse Response Filter (FIRF) together. More specifically, each protein sequence was firstly transformed into the Position Specific Scoring Matrix (PSSM) by exploiting Position Specific Iterated BLAST (PSI-BLAST). Then, to effectively extract the discriminary SIPs feature to improve the performance of SIPs prediction, a FIRF method was used on PSSM. The R’classifier was proposed to execute the classification and predict novel SIPs. We evaluated the performance of the proposed RP-FIRF model and compared it with the state-of-the-art support vector machine (SVM) on *human* and *yeast* datasets, respectively. The proposed model can achieve high average accuracies of 97.89 and 97.35% using five-fold cross-validation. To further evaluate the high performance of the proposed method, we also compared it with other six exiting methods, the experimental results demonstrated that the capacity of our model surpass that of the other previous approaches.

**Conclusion:**

Experimental results show that self-interacting proteins are accurately well-predicted by the proposed model on *human* and *yeast* datasets, respectively. It fully show that the proposed model can predict the SIPs effectively and sufficiently. Thus, RP-FIRF model is an automatic decision support method which should provide useful insights into the recognition of SIPs.

## Background

Protein is a significant component of all cells and tissues of an organism. It is organic macro-molecule or large biological molecule, comprising of many amino acids with different length. It is the basic material of life and the main undertaker of life activity. A number of proteins often associate with their partner or other proteins which is called protein-protein interactions (PPIs) [1]. Self-interacting proteins (SIPs) is a particular type of PPIs, where can interact in terms of duplicate their own genes. SIPs occupy an important role in cellular functions and cellular signal transduction. The majority of chemical reactions occur in living systems which mainly depend on the activity of enzymes. Its essence is a large of protein self-interactions. But it exists a certain difficulty for researchers to discover whether protein can interact with each other or not. The functionality of protein refers to that it could handle the transport of ions and small molecules across cell membranes, depends on their homo-oligomers [2]. In particular, homo-oligomerization can also contribute proteins to compose large structures with increasing error control during synthesis and without increasing genome size [3]. From the past years, many researchers elucidated the overall properties of proteins. Ispolatov *et.al* discovered that the average homodimers of SIPs is more than double the total amount of non-SIPs in the protein interaction networks (PINs) [4]. It is crucial for clarifying the function of SIPs to further understand the regulation of protein function and comprehend whether protein can interact with each other, so that we can better comprehend the mechanism of disease [5]. Liu et al analyzed the properties of SIPs from various aspects information, and applied a logistic regression framework to develop a SIPs prediction model by integrating multiple features [6]. Hence, SIPs will help to improve the stability and prevent the denaturation of a protein via reducing its surface area [7].

So far, a large number of previous methods on the PPIs detection have been proposed [8–10]. For instance, Zhang et al. summarized all sorts of computational methods based on their present knowledge, and proposed an algorithm which integrates structural information with other functional clues [11]. Zou et al. presented a novel fingerprint features and dimensionality reduction strategy for predicting TATA binding proteins, which could improve the prediction accuracy [12]. Hamp et al. introduce a new technique to predict PPIs based on evolutionary profiles and profile-kernel support vector machine [13]. Wan et al. exploited an ensemble multi-label classifier for human protein subcellular location prediction with imbalanced protein source [14]. Song et al. designed a predictor to identify DNA-binding proteins based on unbalanced classification [15]. Sylvain et al. put forward a new PPIs Prediction Engine named PIPE, which is capable of predicting PPIs for any target pair of the *yeast Saccharomyces cerevisiae* proteins from their original structure and without any additional information [16]. Xia et al. presented a sequence-based multi-classifier system that employed autocorrelation descriptor to code an interaction protein pair and chose rotation forest as classifier to infer PPIs [17]. Li et al. provide a scored human PINs with several-fold more interactions and better functional biological relevance than comparable resources by the means of data integration and quality control [18].

However, these approaches could be applied to detect PPIs well [19], but they are not good enough to predict SIPs. Mainly exist in terms of following points: (1) In essence, they also have certain limitations that take the correlation between protein pairs into account for SIPs detection, for example co-expression, co-localization and co-evolution. Nevertheless, these info are of no use for SIPs. (2) The datasets applied to predict PPIs are different from those of SIPs, the datasets of the former are balanced and those of the latter are unbalanced. (3) Besides, prediction of PPIs datasets have no PPIs between same partners. In virtue of reasons, these computational approaches are not suitable for predicting SIPs. Hence, It is becoming more and more significant to exploit an effective calculation method to predict SIPs.

In this paper, we put forward a random projection (RP) bind with Finite Impulse Response Filter (FIRF) model for predicting SIPs from protein sequence information. Furthermore, the main ideas of our raised method includes the following four aspects: (1) The PSI-BLAST could be exploited to convert each protein sequence to a Position Specific Scoring Matrix (PSSM); (2) Employing Finite Impulse Response Filter (FIRF) method to calculate the eigenvalues from protein sequences on a PSSM; (3) To reduce the dimension of feature values which obtained from WT method by applying the Principal Component Analysis (PCA) technique, and removed the noise features from the data, thus the pattern in the data is discovered; (4) RP classifier is applied to build a training set on which the classifiers will be trained. More specifically as follows: first of all, the PSSM of each protein sequence is converted into a 400-dimensional feature vector by employing FIRF method to extract helpful information; then, to remove the influence of noise, we reduced the dimension from 400 to 300 by applying PCA method; At last, realized classification on *yeast* and *human* datasets by relying on RP classifier. The experimental results show that this method outperforms the SVM-based method and other previous methods. It is revealed that the presented method is suitable and perform well for predicting SIPs.

## Results and discussion

### Five-fold cross-validation on *human* and *yeast* datasets

The performance of the proposed method is estimated on the *human* and *yeast* datasets. Aiming at the fairness and over-fitting problems, we repeated the experiment five times on the two same datasets, termed five-fold cross validation. Further, described it in details, we split the *human* dataset which was mainly composed of characteristic values into five non-overlapping pieces, and four parts was randomly chosen as training set and selected the remaining characteristic values as independent test set. Then, we can obtain the results by repeating five times to test our model. To illustrate the rationality, toughness and stability of our algorithm, we also implemented the method of RP-FIRF on the *yeast* dataset.

To guarantee impartiality and objectivity of the test, the parameters for *human* and *yeast* datasets should be set in the same way. In our task, we obtained the better result by adjusting the diverse parameters of RP classifier constantly. Thus, we set the number of blocks B1 = 10 for independent projections to classify the training and test sets, the size of each block was carefully chosen as B2 = 30, and then applying the K-Nearest Neighbor (KNN) base classifier and the leave-one-out test error estimate, where k = seq (1, 30, by = 8).

Afterwards, we test our RP-FIRF prediction method on the two mentioned datasets, and got the results of the two datasets based on 5-fold cross-validation are discovered in Tables 1 and 2. From the Table 1, the data is observed that our proposed method exhibited the five outcomes of average Accuracy (Acc), Sensitivity (Sen), Precision (PE), and Matthews correlation coefficient (MCC) of 97.89, 74.46, 100.00, and 85.31% on *human* dataset and the standard deviations of them of 0.17, 2.18, 0.00, and 1.29%, respectively. Similarly, we can get the results in Table 2 by running experiment on *yeast* dataset, the average Accuracy is 97.35%, average Sensitivity is 77.03%, average Precision is 99.62%, and average MCC is 86.31% and the standard deviations of them of 0.15, 1.17, 0.52, and 0.79%, respectively.

**Table 1:** Results measured by RP-FIRF method on *human* dataset with 5-fold cross-validation

Testing set	Acc (%)	Sen (%)	PE (%)	MCC (%)
1	98.10	76.84	100.00	86.77
2	97.76	74.51	100.00	85.28
3	97.70	71.63	100.00	83.59
4	98.01	73.05	100.00	84.57
5	97.87	76.28	100.00	86.34
Average	97.89 ± 0.17	74.46 ± 2.18	100.00 ± 0.00	85.31 ± 1.29

**Table 2:** Results measured by RP-FIRF method on *yeast* dataset with 5-fold cross-validation

Testing set	Acc (%)	Sen (%)	PE (%)	MCC (%)
1	97.43	78.01	99.10	86.65
2	97.35	77.08	100.00	86.51
3	97.35	75.57	99.00	85.22
4	97.51	78.32	100.00	87.28
5	97.11	76.16	100.00	85.87
Average	97.35 ± 0.15	77.03 ± 1.17	99.62 ± 0.52	86.31 ± 0.79

As mentioned above, It is apparent that our method can receive good effect of SIPs detection because of the appropriate feature extraction and classifier. The presented feature extraction technique plays a critical part in enhancing the calculation accuracy. The specific reasons can be summed up in the following three aspects: (1) PSSM could describe the protein sequence in the form of numerical values. It can be employed to find an amino acid that matches a specific location to give the score in a target protein sequence. Not only can it represents the information of protein sequence, but also it preserves helpful enough information as much as possible. Accordingly, A PSSM contains almost the whole information of one protein sequence for detecting SIPs. (2) Finite impulse response filter (FIRF) feature extraction method of protein sequence can further optimize the performance of our proposed model. (3) To drop the negative influence of noise, PCA was employed to reduce the dimension of data on the condition of the integrity of FIRF feature vector, thus the helpful information in the data will be mined. In a few words, experimental results revealed that our RP-FIRF model is extreme fit for SIPs prediction.

### Compare our proposed model with the SVM-based method

Although the RP-FIRF model achieved accuracy more than 90%, It still needs further test and verify the effectiveness of our presented model. From the point of classification, support vector machine (SVM) is a generalized linear classifier. The SVM-based method has been widely known in many fields of scientific research. Therefore, it’s necessary to compare the prediction accuracy of our RP-FIRF model with the SVM-based method by using the same eigenvalues based on the two above mentioned datasets. We mainly employed the LIBSVM packet tool [20] to implement classification in the experiment. Our first task was to adjust the main parameters of SVM classifier. A radial basis function (RBF) was chosen as the kernel function, and then the two parameters of RBF were adjusted via a grid search algorithm, which were set c = 0.6 and g = 0.02.

As is shown in Tables 3 and 4, we trained and compared the RP-FIRF model with SVM-based model on *yeast* and *human* datasets by employing 5-fold cross-validation respectively. The data from Table 3 can be displayed that the mean of Accuracy, the mean of Sensitivity, the mean of Precision, and the mean of MCC from SVM classifier are 92.32, 32.89, 100.00, and 53.07% on *yeast* dataset, respectively. However, the RP-FIRF method reached 97.35% average Accuracy, 77.03% average Sensitivity, 99.62% average Precision, and 86.31% average MCC on *yeast* dataset. Equally, the data from Table 4 can be shown that the average Accuracy, the average Sensitivity, the average Precision, and the average MCC of SVM classifier are 96.21, 54.44, 100.00, and 72.30% on *human* dataset. Nevertheless, the proposed model achieved 97.89% average Accuracy, 74.46% average Sensitivity, 100.00% average Precision, and 85.31% average MCC on *human* dataset. Stated thus, it is clear that the overall prediction results of RP classifier are much better than those of SVM classifier.

**Table 3:** Comparison results of RP and SVM with FIRF feature vectors on *yeast* dataset

Testing set	Acc (%)	Sen (%)	PE (%)	MCC (%)
SVM + PSSM+FIRF				
1	92.36	32.62	100.00	54.81
2	89.15	6.25	100.00	23.59
3	94.21	45.04	100.00	65.04
4	93.65	44.76	100.00	64.62
5	92.21	35.76	100.00	57.31
Average	92.32 ± 1.96	32.89 ± 15.86	100.00 ± 0.00	53.07 ± 17.08
RP + PSSM+FIRF				
1	97.43	78.01	99.10	86.65
2	97.35	77.08	100.00	86.51
3	97.35	75.57	99.00	85.22
4	97.51	78.32	100.00	87.28
5	97.11	76.16	100.00	85.87
Average	97.35 ± 0.15	77.03 ± 1.17	99.62 ± 0.52	86.31 ± 0.79

**Table 4:** Comparison results of RP and SVM with FIRF feature vectors on *human* dataset

Testing set	Acc (%)	Sen (%)	PE (%)	MCC (%)
SVM + PSSM+FIRF				
1	96.32	55.09	100.00	72.78
2	95.94	53.92	100.00	71.85
3	96.37	55.32	100.00	72.95
4	96.78	56.25	100.00	73.73
5	95.66	51.60	100.00	70.18
Average	96.21 ± 0.43	54.44 ± 1.79	100.00 ± 0.00	72.30 ± 1.36
RP + PSSM+FIRF				
1	98.10	76.84	100.00	86.77
2	97.76	74.51	100.00	85.28
3	97.70	71.63	100.00	83.59
4	98.01	73.05	100.00	84.57
5	97.87	76.28	100.00	86.34
Average	97.89 ± 0.17	74.46 ± 2.18	100.00 ± 0.00	85.31 ± 1.29

Meanwhile, receiver operating characteristic (ROC) curves was applied to analysis the binary classification system (the outcome results only have two categories), was widely applied in many fields such as bioinformatics [21], forecasting of natural hazards [22], machine learning [23], data mining [24] and so on. Therefore, we also used ROC curves to measure the comprehensive index between sensitivity and specificity continuous variable. The area under curves (AUC) could be shown the discriminating capability of the classifier. The closer the top-left corner of the curve is, the higher the prediction accuracy is. Otherwise, the lower the diagnosis result is. In other words, The larger the AUC, the stronger the capability of discernment.

**Fig. 1:**
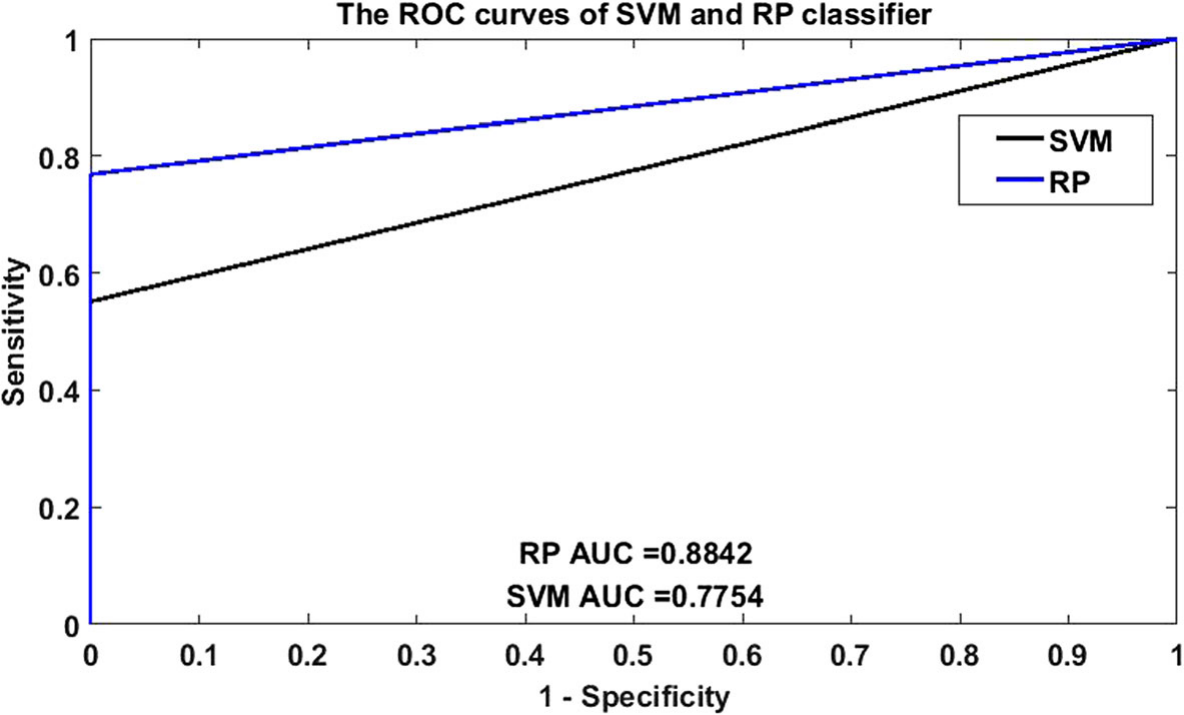
Comparison of ROC curves between RP and SVM on *human* dataset

**Fig. 2:**
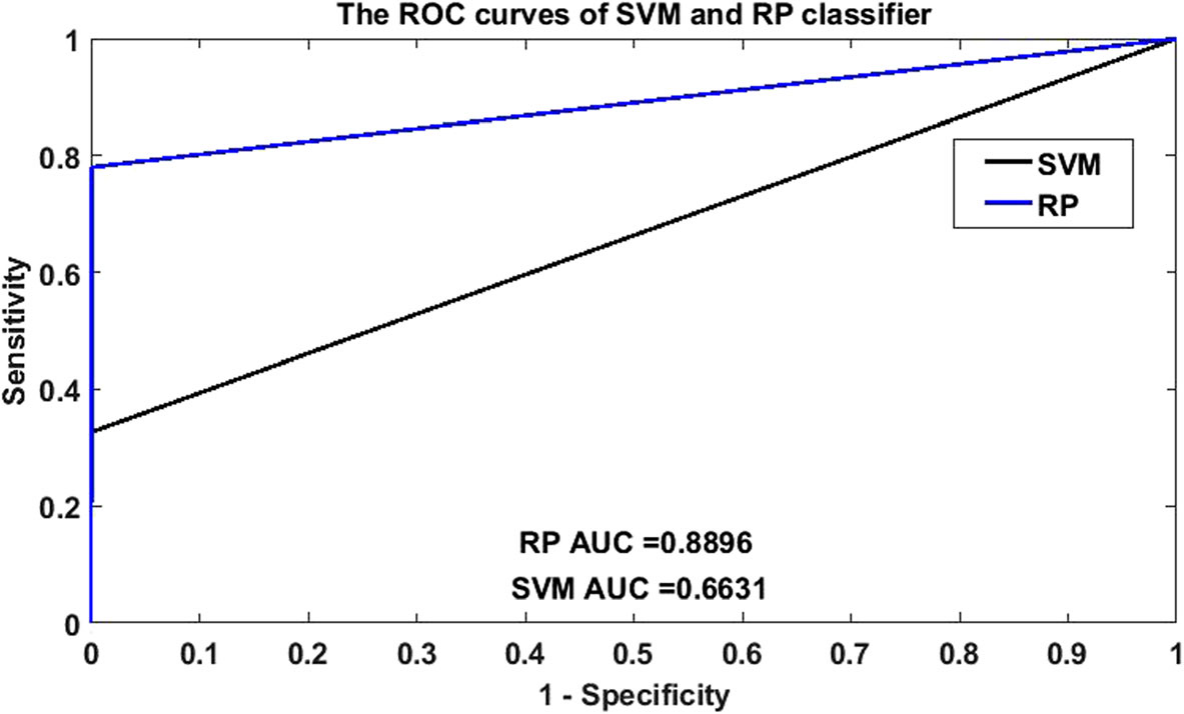
Comparison of ROC curves between RP and SVM on *yeast* dataset

From Fig. 1, we plotted the ROC curves by making a comparison between RP and SVM on *human* dataset, it is clearly that the AUC of SVM classifier is 0.7754 and that of RP classifier is 0.8842. Plots of the RP and SVM classifier on *yeast* dataset in the ROC space are plot in Fig. 2, it is sharply that the AUC of SVM classifier is 0.6631 and that of RP classifier is 0.8896. Anyhow, we demonstrate that the AUC of RP classifier is also significantly larger than that of SVM classifier. So the RP method is an accurate and robust technique for SIPs detection.

### Measure our proposed model against other previous methods

In the process of practice, we measured the quality of proposed model named RP-FIRF with other existing methods based on the two above mentioned datasets to further testify that our approach could obtain better results. We listed a clear statement of account in Tables 5 and 6, which are the comparison results on the two datasets. From Table 5, it is obvious that the RP-FIRF model achieved the highest average accuracy of 97.35% than the other six methods (range from 66.28 to 87.46%) on *yeast* dataset. At the same instant, it is clear to see that the other six methods got lower MCC (range from 15.77 to 28.42%) than our proposed model of 86.31% on the same dataset. In exactly the same way, from Table 6, the overall results of our prediction approach is also outperform the other six methods on *human* dataset. To make a summary of it, we measured our RP-FIRF model against with the other six approaches on *yeast* and *human* datasets respectively, the prediction accuracy of the overall experimental results can be improved. This fully illustrates that a good feature extraction tool and a suitable classifier is very important for predicting model. It is further illustrated that our method is superior to the other six approaches and quite suitable for SIPs preditcion.

**Table 5:** Performance results between RP-FIRF model and the other methods on *yeast* dataset

Model	Acc (%)	Sp (%)	Sen (%)	MCC (%)	AUC
SLIPPER [6]	71.90	72.18	69.72	28.42	0.7723
DXECPPI [25]	87.46	94.93	29.44	28.25	0.6934
PPIevo [26]	66.28	87.46	60.14	18.01	0.6728
LocFuse [27]	66.66	68.10	55.49	15.77	0.7087
CRS [28]	72.69	74.37	59.58	23.68	0.7115
SPAR [28]	76.96	80.02	53.24	24.84	0.7455
Proposed method	97.35	99.96	77.03	86.31	0.8896

**Table 6:** Performance results between RP-FIRF model and the other methods on *human* dataset

Model	Acc (%)	Sp (%)	Sen (%)	MCC (%)	AUC
SLIPPER [6]	91.10	95.06	47.26	41.97	0.8723
DXECPPI [25]	30.90	25.83	87.08	8.25	0.5806
PPIevo [26]	78.04	25.82	87.83	20.82	0.7329
LocFuse [27]	80.66	80.50	50.83	20.26	0.7087
CRS [28]	91.54	96.72	34.17	36.33	0.8196
SPAR [28]	92.09	97.40	33.33	38.36	0.8229
Proposed method	97.89	100.00	74.46	85.31	0.8842

## Conclusion

In the study, a machine learning model was put forward to predict SIPs which based on protein primary sequence. This model was developed by combining Finite Impulse Response Filter with Random Projection classifier, which was termed RP-FIRF. The mainly improvements for this method are attributable to the following aspects: (1) A reasonable representative method FIRF is used to effectively extract the discriminary features, which can process and analyze protein sequence data well. (2) The RP classifier is strongly suitable for predicting SIPs, and a high recognition accuracy can be obtained. The experimental results measured by the presented model on *yeast and human* datasets revealed that the performance of RP method is significantly better than that of the SVM-based method and other six previous methods. It fully shows that the integration of FIRF method with RP classifier is able to significantly improve the accuracies of SIPs prediction. Overall, we have predicted a reliable set of SIPs suitable for further computational as well as experimental analyses. For the future research, there will be more and more effective feature extraction methods and machine learning approaches exploited for detecting SIPs.

## Materials and methodology

### Datasets

In our study, we constructed the datasets mainly derived from the UniProt database [29] which contains 20,199 curated *human* protein sequences. There are many different types of resources such as DIP [30], BioGRID [31], IntAct [32], InnateDB [33] and MatrixDB [34], we can get the PPIs related information from them. In relational databases, we mainly set up the datasets for SIPs which embodies two identical interacting protein sequences and whose type of interaction was characterized as “direct interaction”. Based on that, we can construct the datasets for the experiment by applying 2994 *human* self-interacting protein sequences.

For the 2994 *human* SIPs, we need to single out the datasets for the experiment and assess the performance of the RP-FIRF model, which mainly includes three steps [28]: (1) If the protein sequences which may be fragments, we will remove it and retain the length of protein sequences between 50 residues and 5000 residues from all the *human* proteome; (2) To build up the positive dataset of *human*, we formed a high-grade SIPs data which should meet one of the following conditions: (a) the self-interactions were revealed by at least one small-scale experiment or two sorts of large-scale experiments; (b) the protein has been announced as homo-oligomer (containing homodimer and homotrimer) in UniProt; (c) it has been reported by more than two publications for self-interactions; (3) For the *human* negative dataset, we removed the whole types of SIPs from all the *human* proteome (contains proteins annotated as ‘direct interaction’ and more extensive ‘physical association’) and SIPs detection in UniProt database. To sum it up, we obtained the ultimate *human* dataset for the experiment which was mainly composed of 1441 SIPs and 15,938 non-SIPs [28].

Just as the construction of *human* dataset, we also further assess the cross-species ability of the RP-FIRF model by repeating the same strategy mentioned above to generate the *yeast* dataset. Finally, 710 SIPs was assigned to form the *yeast* positive dataset and 5511 non-SIPs was allocated to constitute the *yeast* negative dataset [28].

### Assessment tools

In the field of machine learning, confusion matrix is always employed in evaluating the classification model, also known as an error matrix [35, 36]. It indicates information about actual and predicted classifications for two class classifier which could be shown as the follow Table 7.

**Table 7:** Confusion Matrix

		Predict	
		Negative	Positive
Actual	Negative	TN	FN
	Positive	FP	TP

In our study, in the interest of size up the steadiness and effectiveness of our present model, we computed the values of 5 parameters: Accuracy (Acc), Sensitivity (Sen), specificity (Sp), Precision (PE) and Matthews’s Correlation Coefficient (MCC), respectively. These parameters can be described as follows:

1 $$ Acc=\frac{TP+ TN}{TP+ FP+ TN+ FN} $$

2 $$ Sen=\frac{TP}{TP+ FN} $$

3 $$ Sp=\frac{TN}{FP+ TN} $$

4 $$ PE=\frac{TP}{FP+ TP} $$

5 $$ MCC=\frac{\left( TP\times TN\right)-\left( FP\times FN\right)}{\sqrt{\left( TP+ FN\right)\times \left( TN+ FP\right)\times \left( TP+ FP\right)\times \left( TN+ FN\right)}} $$

where, *TP* (i.e. true positives) is the quantity of true interacting pairs correctly predicted. *FP* (i.e. false positives) represents the number of true non-interacting pairs falsely predicted. *TN* (i.e. true negatives) is the count of true non-interacting pairs predicted correctly. *FN* (i.e. false negatives) represents true interacting pairs falsely predicted to be non-interacting pairs. On the basis of these parameters, a ROC curve was plotted to evaluate the performance of random projection method. And then, we can calculate the area under curve (AUC) to measure the performance of the classifier.

### Position specific scoring matrix

In our experiment, Position Specific Scoring Matrix (PSSM) is a helpful technique which was employed to detect distantly related proteins [37]. Accordingly, each protein sequence information was transformed into PSSM by using the PSI-BLAST [38]. And then, a given protein sequence can be converted into an *H* × 20 PSSM which could be represented as follow:

6 $$ M=\left\{ M\alpha \kern0.1em \beta \kern0.3em \alpha :1=1\cdots H,\beta =1\cdots 20\right\} $$

where *H* denotes the length of a protein sequence, and *20* is the number of amino acids due to every sequence was constituted by 20 different amino acids. For the query protein sequence, the score *C*_*αβ*_ indicates that the *β-th* amino acid in the position of *α* assigned from a PSSM. Therefore, *C*_*αβ*_ could be described as:

7 $$ C\alpha \beta ={\sum}_{k=1}^{20}p\left(\alpha, k\right)\times q\left(\beta, k\right) $$

where *p(α,k)* represents the occurrence frequency of the *k-th* amino acid at location of *α*, and *q(β,k)* is the Dayhoff’s mutation matrix value between *β-th* and *k-th* amino acids. In addition, diverse scores determine different relative location relationships, a greater degree means a strongly conservative position, and otherwise a weakly conservative position can gain a lower value.

Overall, PSSM has been more and more important in the research of SIPs prediction. In a detailed and exact way, we employed PSI-BLAST to obtain the PSSM from each protein sequence for detecting SIPs. To achieve a better score and a large scale of homologous sequences, the *E*-value parameter of PSI-BLAST was set to be 0.001 which reported for a given result represents the quantity of two sequences’ alignments and selected three iterations in this experiment [39, 40]. Afterwards we can achieve a 20-dimensional matrix which consists of *M × 20* elements based on PSSM, where *M* represents the count of residues of a protein, and *20* denote the 20 types of amino acids.

### Finite impulse response filters

In the field of digital signal processing (DSP) [41], finite impulse response filter (FIRF) is one of the most commonly used components, which can perform the function of signal pre-modulation and frequency band selection and filtering. FIRF are widely employed in many fields such as communications [42], image processing [43], pattern recognition [44], wireless sensor network [45] and so on. Many methods of DSP were applied in the fundamental research of cytology, brain neurology, genetics and other fields. In our work, we applied FIRF to process the characteristics of protein sequences, which would be used to predict the SIPs. Therefore, many important features of the problem can be fully highlighted by the FIRF method, and then it could devote to the details of the problem. We design it by using Fourier series method in details as follows.

At first, the corresponding Frequency Response Function of FIRF transfer function can be described as:

8 $$ H\left({e}^{jw}\right)=\sum \limits_{n=0}^{N-1}h(n){e}^{- jwn} $$

where, *h(n)* is the available impulse response sequence, and *N* represents the sample sizes of frequency response *H (e*^*jw*^*)*. Given the frequency response *H*_*d*_ *(e*^*jw*^*)* of ideal filter, and let *H (e*^*jw*^*)* approach *H*_*d*_ *(e*^*jw*^*)* infinitely.

9 $$ {H}_d\left({e}^{jw}\right)=\sum \limits_{n=-\infty}^{\infty }{h}_d(n){e}^{- jwn} $$

And then, we can achieve the *-h*_*d*_*(n)* by employing inverse Fourier transform of *H*_*d*_ *(e*^*jw*^*).* The *h*_*d*_*(n)* is built as

10 $$ {h}_d(n)=\frac{1}{2\pi }{\int}_{-\pi}^{\pi }{H}_d\left({e}^{jw}\right){e}^{jw n} dw $$

where *h*_*d*_*(n)* is a finite length. If *h*_*d*_*(n)* is an infinite length, we can intercept *h*_*d*_*(n)* by applying a finite length of the windows function sequence *w(n)*.

11 $$ h(n)={h}_d(n)w(n) $$

According to the above formula, we can gain the unit sample response for our designed FIR filter. To check the filter whether meet the design requirements by follow formula.

12 $$ H\left({e}^{jw}\right)= DTFT\left[h(n)\right] $$

The integral square error (ISE) between the frequency response of ideal filter and our designed filter can be defined as follow:

13 $$ {\varepsilon}^2=\frac{1}{2\pi }{\int}_{-\pi}^{\pi }{\left|{H}_d\left({e}^{jw}\right)-H\left({e}^{jw}\right)\right|}^2 dw $$

In our study, we cannot directly extract the eigenvalues from the protein because of each protein sequence have the different amino acids composition. To prevent the generation of unequal lengths of feature vectors, we multiply the transpose of PSSM by PSSM to achieve *20 × 20* matrix. and then, we employ the FIRF technique to transform the PSSM of each protein sequence into a feature vector which have the same size with *20 × 20* matrix. Afterwards, these feature values could be computed as a 400-dimensional vector. Eventually, every protein sequence from the two above mentioned datasets was transformed into a 400-dimensional vector by employing FIRF approach.

For the sake of remove the influence of noise and improve the result of SIPs prediction, we applied the Principal Component Analysis (PCA) to remove the influence of noisy features on the two above mentioned datasets. So as to we can reduce the dimension of the two datasets from 400 to 300. Accordingly, we could employ a small number of information to represent the whole data and push the complexity into smaller, so as to improve the generalization error.

### Random projection classifier

In mathematics and statistics, Random Projection (RP) is a classifier for dimensionality reduction of some points which lie in Euclidean space. RP classifier showed that *N* points in *N* dimensional space can almost always be mapped to a space of dimension *ClogN* with command on the ratio of error and distances [46, 47]. It has been successfully applied in rebuilding of frequency-sparse signals [48], face recognition [49], protein subcellular localization [50] and textual and visual information retrieval [51].

We formally describe the RP classifier as follow in details. At first, let

14 $$ \varGamma ={\left\{ Ai\right\}}_{i=1}^N, Ai\in {R}^n $$

be the primitive high dimensional space dataset, where *n* represents the high dimension and *N* denotes the number of the dataset. The goal of dimensionality reduction is embedding the vectors into a lower dimensional space *R*^*q*^ from a high dimension *R*^*n*^, where *q < <n*. The output of data is defined as follow:

15 $$ \overset{\sim }{\varGamma }={\left\{\overset{\sim }{A_i}\right\}}_{i=1}^N,\overset{\sim }{A_i}\in {R}^q $$

where *q* is close to the intrinsic dimensionality of *Γ*. Thus, the vectors of *Γ* was regarded as embedding vectors.

If we want to reduce the dimension of *Γ* via random projection method, a random vector set *γ = {r*_*i*_*} k i = 1* must be constructed at first, where *r*_*i*_*∈R*^*q*^. The random basis can be obtained by two common choices as follow [46]:

1. The vectors *{r*_*i*_*} k i = 1* are normally distributed over the *q* dimensional unit sphere.
2. The components of the vectors *{r*_*i*_*} k i = 1* are chosen Bernoulli + 1/− 1 distribution and the vectors are standardized so that *||r*_*i*_*||*_*l2*_ *= 1* for *i = 1, …,n*.

Then, the columns of *q × n* matrix *R* are consisted of the vectors in *γ*. The embedding result *Ã*_*i*_ of *A*_*i*_ can be got by

16 $$ \overset{\sim }{A_i}=R\cdot {A}_i $$

In our proposed method, random projection classifier will be trained on a training set. And we enrich the component of the ensemble method based on random projection.

Next, the size of target space was set to a part of around the space where the training members reside. We built a size of *n × N* matrix *G* whose columns are made up the column vectors in *Γ*. The training set *Γ* have given in Eq.14.

17 $$ G=\left({A}_1|{A}_2|...|{A}_N\right) $$

Then, we construct *k* random matrices *{R*_*i*_*} k i = 1* whose size is *q × n*, *q* and *n* are introduced in the above mentioned paragraph, and *k* is the quantity of classifiers. Here, the columns of matrices are normalized so as to the *l*_*2*_ norm is 1.

And then, in our method, to construct the training sets *{T*_*i*_*} k i = 1* by projecting *G* onto *{R*_*i*_*} k i = 1* which is the *k* random matrices. It can be represented as follow:

18 $$ {T}_i={R}_i\cdot G,\kern0.5em i=1,...,k $$

The training sets are imported into an inducer and the export results are a piece of classifiers *{ℓ*_*i*_*} k i = 1*. How to classify a new dataset *I* through classifier *ℓ*_*i*_. At first, we embed *I* into the dimensionality reduction space *R*^*q*^. Then, It can be owned via mapping *u* to the random matrix *R*_*i*_ as follow:

19 $$ \overset{\sim }{I}={R}_i\cdot I $$

where *Ĩ* is the inlaying of *u*, the classification of *Ĩ* can be garnered from the classification of *I* by *ℓ*_*i*_. In this ensemble method, the random projection classifier use a data-driven voting threshold which is employed to classification outcomes of the whole classifiers *{ℓ*_*i*_*} k i = 1* for the *Ĩ* to decide produce the ultimate classification result of *Ĩ*.

In this experiment, the random projections were split up non-overlapping blocks where B1 = 10 and each one carefully chosen from a block of size B2 = 30 that achieved the smallest estimate of the test error. We used the k-Nearest Neighbor (KNN) as base classifier and the leave-one-out test error estimate, where k = seq (1, 30, by = 8). The prior probability of interaction pairs in the training sample set was taken as the voting parameter. Our classifier integrates the results of taking advantage of the base classifier on the selected projection, with the data-driven voting threshold to confirm the final mission.

## Data Availability

Not applicable.
